# Commentary: Effect of fecal microbiota transplantation on non-alcoholic fatty liver disease: A randomized clinical trial

**DOI:** 10.3389/fcimb.2022.1056394

**Published:** 2022-11-10

**Authors:** Jieyi Wang, Jinhan Chen, Mingxian Chen

**Affiliations:** ^1^ Department of Gastroenterology, Tongde Hospital of Zhejiang Province, Hangzhou, China; ^2^ The Second Affiliated College Of Zhejiang Chinese Medical University, Hangzhou, China

**Keywords:** fecal microbiota transplantation (FMT), non-alcoholic fatty liver disease (NAFLD), gut microbiota, exercise therapy, diet therapy long-term efficacy, lifestyle interventions

## Introduction

A study entitled “Effect of Fecal Microbiota Transplantation on Non-Alcoholic Fatty Liver Disease: A Randomized Clinical Trial” was recently published in Frontiers in Cellular and Infection Microbiology by (Xue et al., 2022). According to the summary of the respective article, fecal microbiota transplantation (FMT) can reduce hepatic fat accumulation by improving gut microbiota dysbiosis. Based on the participant’s preferences, individuals with non-alcoholic fatty liver disease (NAFLD) were included in the study and randomly assigned to either of the two groups: FMT or traditional treatment. The feces needed for the FMT group’s therapy as well as the healthy group’s feces were provided by healthy volunteers from Guangdong Pharmaceutical University. When L. Xue et al. examined the gut microbiota’s features and alterations, they discovered that patients with NAFLD before to FMT had lower Chaol indices [prior to FMT (pri-FMT) vs. healthy group, *p* < 0.05] than healthy people, indicating a reduced abundance of the gut microbiota in NAFLD patients. In this case, there is no baseline data comparison between the healthy group and the pri-FMT group.

NAFLD is currently thought to be the hepatic manifestation of the metabolic syndrome and is frequently linked to metabolic risk factors such obesity, dyslipidemia, hypertension, and diabetes (Buzzetti et al., 2016). However, not all NAFLD patients have the same disease drivers; for instance, some people may have disease that is primarily brought on by lipid dysregulation, while others may have disease that is brought on by increased inflammation or insulin resistance, or by a combination of host genetics, the microbiome, or other determinants (Loomba et al., 2021). Numerous studies have shown that the gut microbiota is crucial in the pathogenesis of NAFLD. (Boursier et al., 2016; Leung et al., 2016; Canfora et al., 2019; Aron-Wisnewsky et al., 2020; Tilg et al., 2020). Age, diet, exercise can also change gut microbiota, and all above can cause favorable changes in the structure and functions of the gut microbiota (Hasan and Yang, 2019; Gubert et al., 2020; Liu et al., 2020; Mohr et al., 2020). Thus, changing age, different diet and exercise habits were all involved in modulating gut microbiota. With age, there is a broad shift in the types of gut microbiota. For example, the Firmicutes phylum have the opposite tendency from those of the Bacteroidetes phylum, which tend to dominate numerically in youth but drastically fall in numbers as they age (Conlon and Bird, 2014). Obviously, there was a significant age difference between pri-FMT group and healthy group. For undergraduate students in the healthy group are fairly young, but the average age of pri-FMT group patients at around 57 years (**Table 1**), which greatly affected Chaol indexes of the gut microbiota. In addition, the usual lifestyle habits, including exercise and diet, also affect the abundance of gut microbiota, which need to be given in the baseline information comparison table between pri-FMT group and healthy group.

**Table 1:** Baseline comparison between FMT group and non-FMT group.

Characteristic	FMT group (n=47)		Non-FMT group (n=28)		*P value*
	Before treatment	After treatment	Before treatment	After treatment	
Gender (male)	25 (53.2%)		14 (50%)		>0.05
Age	57.3 ± 13.4		60.2 ± 8.5		>0.05
Hypertension	23 (48.9%)		12 (42.8%)		>0.05
Type 2 diabetes	15 (31.9%)		8 (28.5%)		>0.05
BMI (kg/m^2^)	27.7 ± 4.5	27.4 ± 4.4	28.0 ± 5.2	28.4 ± 5.2	>0.05
PLT (10×10^9)	231.8 ± 66.7	229.8 ± 60.1	221.4 ± 73.7	223.9 ± 75.6	>0.05
Hb (g/L)	137.9 ± 13.6	139.5 ± 14.4	134.6 ± 16.5	137.6 ± 12.8	>0.05
Fasting blood glucose (mmol/L)	5.3 ± 1.2	5.3 ± 1.3	5.6 ± 1.6	5.8 ± 1.9	>0.05
Fasting insulin (mU)	15.1 ± 8.9	13.4 ± 6.7	12.6 ± 6.0	15.8 ± 8.9	>0.05
HOMA IR	3.8 ± 2.9	3.4 ± 2.2	3.2 ± 1.7	4.5 ± 3.3	>0.05
Uric acid (umol/L)	366.5 ± 84.1	370.8 ± 81.3	419.5 ± 93.1	399.8 ± 81.3	>0.05

FMT, fecal microbiota transplantation; BMI, body mass index; PLT, platelets; Hb, hemoglobin; HOMA IR, homeostasis model assessment of insulin resistance. P, baseline FMT vs baseline non-FMT.

Second, exercise variables (type/frequency/intensity/duration) were not all specified in the research design. It has been reported in the researches that different exercise lead to significantly differentiated abundances of the gut microbiota (Morita et al., 2019; Ticinesi et al., 2019), liver function and biochemical parameters in NAFLD patients (Golabi et al., 2016; Hashida et al., 2017; Zhou et al., 2021). In this study, only more than 40 minutes of exercise per day were recommended; the intensity and type of exercise were not specified. In practice, patient comprehension biases and varying exercise preferences may have an impact on the accuracy of the results. Therefore, the study should clarify both the type and intensity of exercise.

Third, despite the fact that there are numerous potential pathways into gut microbiota modification in NAFLD patients, this study did not evaluate the long-term efficacy of FMT, which is required for assessing the treatment potential for NAFLD. FMT treatment may be an effective supplement to NAFLD management, but more research is needed to identify its maximum validity.

Scientific knowledge regarding diversity and richness of gut microbiota in Patients with NAFLD is still being explored. Rapid progress in this field will be dependent on the falsifiability of hypotheses based on rigorous scientific evidence. We believe that the findings reported by L. Xue and colleagues will help to achieve this goal, either directly or indirectly; however, this report needs to be more rigorous in designing experimental protocols, to clarify that NAFLD is an independent factor contributing to gut microbiota disorders, and to determine the precise, long-term efficacy of FMT in treating NAFLD.

## Author contributions

JW and JC wrote the manuscript with the support from MC. The original idea was conceived by MC. All authors contributed to the article and approved the submitted version.

## Funding

This work was funded by 2022 Special Project for Modernization of Chinese Medicine in Zhejiang Province (No. 2022ZX001), Zhejiang Province 551 Health Talent Training Project(Zhejiang Provincial Health and Health Commission Office [2021] 40).

## Conflict of interest

The authors declare that the research was conducted in the absence of any commercial or financial relationships that could be construed as a potential conflict of interest.

## Publisher’s note

All claims expressed in this article are solely those of the authors and do not necessarily represent those of their affiliated organizations, or those of the publisher, the editors and the reviewers. Any product that may be evaluated in this article, or claim that may be made by its manufacturer, is not guaranteed or endorsed by the publisher.

## References

[B1] Aron-Wisnewsky J. Vigliotti C. Witjes J. Le P. Holleboom A. G. Verheij J.. (2020). Gut microbiota and human NAFLD: disentangling microbial signatures from metabolic disorders. Nat. Rev. Gastroenterol. Hepatology. 17, 279–297. doi: 10.1038/s41575-020-0269-9 32152478

[B2] Boursier J. Mueller O. Barret M. Machado M. Fizanne L. Araujo-Perez F.. (2016). The severity of nonalcoholic fatty liver disease is associated with gut dysbiosis and shift in the metabolic function of the gut microbiota. Hepatology. 63, 764–775. doi: 10.1002/hep.28356 26600078 PMC4975935

[B3] Buzzetti E. Pinzani M. Tsochatzis E. A. (2016). The multiple-hit pathogenesis of non-alcoholic fatty liver disease (NAFLD). Metabolism. 65, 1038–1048. doi: 10.1016/j.metabol.2015.12.012 26823198

[B4] Canfora E. E. Meex R. C. R. Venema K. Blaak E. E. (2019). Gut microbial metabolites in obesity, NAFLD and T2DM. Nat. Rev. Endocrinology. 15, 261–273. doi: 10.1038/s41574-019-0156-z 30670819

[B5] Conlon M. A. Bird A. R. (2014). The impact of diet and lifestyle on gut microbiota and human health. Nutrients. 7, 17–44. doi: 10.3390/nu7010017 25545101 PMC4303825

[B6] Golabi P. Locklear C. T. Austin P. Afdhal S. Byrns M. Gerber L.. (2016). Effectiveness of exercise in hepatic fat mobilization in non-alcoholic fatty liver disease: Systematic review. World J. Gastroenterol. 22, 6318–6327. doi: 10.3748/wjg.v22.i27.6318 27468220 PMC4945989

[B7] Gubert C. Kong G. Renoir T. Hannan A. J. (2020). Exercise, diet and stress as modulators of gut microbiota: Implications for neurodegenerative diseases. Neurobiol. Disease. 134:104621. doi: 10.1016/j.nbd.2019.104621 31628992

[B8] Hasan N. Yang H. (2019). Factors affecting the composition of the gut microbiota, and its modulation. PeerJ. 7:e7502. doi: 10.7717/peerj.7502 31440436 PMC6699480

[B9] Hashida R. Kawaguchi T. Bekki M. Omoto M. Matsuse H. Nago T.. (2017). Aerobic vs. resistance exercise in non-alcoholic fatty liver disease: A systematic review. J. Hepatol. 66, 142–152. doi: 10.1016/j.jhep.2016.08.023 27639843

[B10] Leung C. Rivera L. Furness J. B. Angus P. W. (2016). The role of the gut microbiota in NAFLD. Nat. Rev. Gastroenterol. Hepatology. 13, 412–425. doi: 10.1038/nrgastro.2016.85 27273168

[B11] Liu Y. Wang Y. Ni Y. Cheung C. K. Y. Lam K. S. L. Wang Y.. (2020). Gut microbiome fermentation determines the efficacy of exercise for diabetes prevention. Cell Metab. 31, 77–7+. doi: 10.1016/j.cmet.2019.11.001 31786155

[B12] Loomba R. Friedman S. L. Shulman G. I. (2021). Mechanisms and disease consequences of nonalcoholic fatty liver disease. Cell. 184, 2537–2564. doi: 10.1016/j.cell.2021.04.015 33989548 PMC12168897

[B13] Mohr A. E. Jaeger R. Carpenter K. C. Kerksick C. M. Purpura M. Townsend J. R.. (2020). The athletic gut microbiota. J. Int. Soc. Sports Nutr. 17:1–33. doi: 10.1186/s12970-020-00353-w PMC7218537 32398103

[B14] Morita E. Yokoyama H. Imai D. Takeda R. Ota A. Kawai E.. (2019). Aerobic exercise training with brisk walking increases intestinal bacteroides in healthy elderly women. Nutrients. 11:868. doi: 10.3390/nu11040868 30999699 PMC6520866

[B15] Ticinesi A. Nouvenne A. Cerundolo N. Catania P. Prati B. Tana C.. (2019). Gut microbiota, muscle mass and function in aging: A focus on physical frailty and sarcopenia. Nutrients. 11:e1633. doi: 10.3390/nu11071633 PMC6683074 31319564

[B16] Tilg H. Zmora N. Adolph T. E. Elinav E. (2020). The intestinal microbiota fuelling metabolic inflammation. Nat. Rev. Immunol. 20, 40–54. doi: 10.1038/s41577-019-0198-4 31388093

[B17] Xue L. Deng Z. Luo W. He X. Chen Y. (2022). Effect of Fecal Microbiota Transplantation on Non-Alcoholic Fatty Liver Disease: A Randomized Clinical Trial. Front Cell Infect Microbiol. 12, 759306. doi: 10.3389/fcimb.2022.759306 35860380 PMC9289257

[B18] Zhou B. J. Huang G. Wang W. Zhu L. H. Deng Y. X. He Y. Y.. (2021). Intervention effects of four exercise modalities on nonalcoholic fatty liver disease: a systematic review and Bayesian network meta-analysis. Eur. Rev. Med. Pharmacol. Sci. 25, 7687–7697. doi: 10.26355/eurrev_202112_27615 34982430

